# Holistic Vehicle Instrumentation for Assessing Driver Driving Styles

**DOI:** 10.3390/s21041427

**Published:** 2021-02-18

**Authors:** María Garrosa, Ester Olmeda, Sergio Fuentes del Toro, Vicente Díaz

**Affiliations:** 1 Department of Mechanical Engineering, Universidad Carlos III de Madrid, Avda. de la Universidad 30, 28911 Leganés, Spain; eolmeda@ing.uc3m.es (E.O.); sfuentes@ing.uc3m.es (S.F.d.T.); vdiaz@ing.uc3m.es (V.D.); 2 Institute for Automotive Vehicle Safety (ISVA), Universidad Carlos III de Madrid, Avda. de la Universidad 30, 28911 Leganés, Spain

**Keywords:** pressure sensor, driving style, autonomous vehicle, types of braking

## Abstract

Nowadays, autonomous vehicles are increasing, and the driving scenario that includes both autonomous and human-driven vehicles is a fact. Knowing the driving styles of drivers in the process of automating vehicles is interest in order to make driving as natural as possible. To this end, this article presents a first approach to the design of a controller for the braking system capable of imitating the different manoeuvres that any driver performs while driving. With this aim, different experimental tests have been carried out with a vehicle instrumented with sensors capable of providing real-time information related to the braking system. The experimental tests consist of reproducing a series of braking manoeuvres at different speeds on a flat floor track following a straight path. The tests distinguish between three types of braking manoeuvre: maintained, progressive and emergency braking, which cover all the driving circumstances in which the braking system may intervene. This article presents an innovative approach to characterise braking types thanks to the methodology of analysing the data obtained by sensors during experimental tests. The characterisation of braking types makes it possible to dynamically classify three driving styles: cautious, normal and aggressive. The proposed classifications allow it possible to identify the driving styles on the basis of the pressure in the hydraulic brake circuit, the force exerted by the driver on the brake pedal, the longitudinal deceleration and the braking power, knowing in all cases the speed of the vehicle. The experiments are limited by the fact that there are no other vehicles, obstacles, etc. in the vehicle’s environment, but in this article the focus is exclusively on characterising a driver with methods that use the vehicle’s dynamic responses measured by on-board sensors. The results of this study can be used to define the driving style of an autonomous vehicle.

## 1. Introduction

During the next decade the autonomous vehicles will be released to the market. For user acceptance, these vehicles must not only be safe and reliable, but also provide a comfortable and safe driver experience. However, individual perceptions of comfort can vary considerably among users. While some users may prefer sporty driving with high acceleration, others may prefer a comfortable style. Although comfort is a subjective task and it is influenced by driving style [1], some studies have already shown that the users would prefer that an autonomous vehicle drives similar to their own driving style [2,3]. Another example is the study developed by Yusof, N. M et al. [4] where an experiment carried out with an equipped car was conducted to investigate which driving style for an autonomous vehicle is preferred by experimental drivers. Previously, the experimental drivers were identified as assertive or defensive by means of a questionnaire. For these two types of drivers (assertive and defensive) three different simulations of autonomous driving are proposed by modifying the acceleration in the longitudinal, lateral and vertical direction. The results showed that the defensive driving style was preferred and there were variations between participants related to their own driving style. In [5] the proposed system provides a personalized autonomous self-driving style like a human driver. The simulation and experimental results demonstrated that the proposed system can manoeuvre the self-driving vehicle like a human driver by tracking the specified criterion of admissible acceleration and jerk. In [6] the system evaluated two different types of driving manoeuvres: overtaking and deceleration at an intersection. The user can select from a set of four predefined driving styles: defensive, normal, assertive and a specific style with very low accelerations and decelerations called light rail transit. A defensive style that should increase comfort and perceived safety. A normal style, which represents the average preferences of all study participants and an assertive style, for users who want the most efficient way of transportation and have high trust in the safe functioning of the vehicle. Therefore, it is essential the recognition of driving style and driver intention inference for the integration and development of the autonomous vehicles.

Today’s breakthrough in the automotive industry is due, in terms of safety, to the continuous improvement of intelligent driver assistance systems. This boom is the result of manufacturers’ awareness and their aim to build safer vehicles in order to reduce the number of road fatalities. These systems interact with the driver to help them practice safer driving, taking control of the vehicle if necessary. Since most road accidents are caused by the human driver, the advancement of these assistance techniques reinforces the increase in passenger safety and comfort. Driving style plays an important role in driving safety. Furthermore, it is key for advance driver assistance systems (ADAS) development, toward increasing levels of vehicle automation. This fact has motivated numerous research and development efforts on driving style identification and classification [7,8,9,10]. ADAS would benefit from knowledge of driving styles to predict and anticipate driver reactions and adjust to individual users. Advanced driving assistance systems can be adapted to measure driving styles in real-time and then provide the driver with feedback based upon driving style information. In [11], by using a smartphone-based ADAS for navigation and collision warning, a positive change in the behaviour of the driver who adopts a more defensive driving style is achieved in real time. The relationship between drivers and ADAS is an important aspect to consider, and more specifically, how ADAS can adapt to the characteristics of each driver. A model for incorporating human factors into an ADAS is proposed in [12]. Other safety-related applications where driving style awareness is of interest include driving fatigue detection [13] and distraction detection [14].

Motivated by the previous review, this paper provides an innovative approach to dynamically classify driving styles by means of signals related to driver behaviour (brake pedal force) and vehicle state (speed, acceleration and brake circuit pressure).

This article presents a study that was carried out thanks to the participation of different volunteers and the driving event that was studied is the braking manoeuvre. The database proposed in this article has 267 experiments. The experiments consisted in reproducing a set of braking manoeuvres at different speeds on a flat floor track following a straight path. The test speeds were 20, 30, 40, 40, 50, 60, 70 and 80 km/h. To achieve the objective of this article, which is to establish classifications of driving styles, the vehicle was instrumented with different sensors and three braking manoeuvres are proposed for the execution of the experiments: maintained, progressive and emergency. The sensors fitted to the vehicle were a pressure sensor incorporated in the hydraulic circuit of the braking system, a load cell installed in the brake pedal, an Inertial Measurement Unit (IMU) in the centre of gravity of the vehicle and a Global Position System (GPS) receiver. The signals from these sensors provided information on the pressure in the brake circuit, the force exerted by the driver on the brake pedal, deceleration and vehicle speed. The research focuses on the potential of these sensors to obtain information on the parameters chosen to characterise the different types of braking. Thanks to the novel methodology used to analyse the sensor signals, it has been possible to establish different classifications for three driving styles: cautious, normal and aggressive, based on each of the variables measured by the sensors.

The manuscript is organised as follows: Section 2 reviews the sensors and parameters measured to establish driving style classifications, as well as the different approaches; Section 3 explains the vehicle instrumentation and the methodology used to perform the experimental tests; Section 4 presents the experimental results and the driving style classifications; Section 5 concludes the paper.

## 2. Related Works

According to the literature reviewed, the sensors used to obtain the parameters for characterizing driving styles are part of the equipment of the vehicles currently on the market and serve as support for the security systems they incorporate, thus avoiding the installation of new sensors. The sensor that has been most used is the IMU [15,16,17,18]. It is also widespread the use of Smartphones or tablets instead of the conventional sensors of CAN-bus vehicles. Smartphones include accelerometers, cameras, GPS [19], gyroscopes and geomagnetic field sensors [20]. Table 1 presents the sensors that have been used to identify driving styles [7].

The first step to identify driving styles is to determine the parameters that allow a robust classification to be made. In the literature, depending on the application for which the driving style classification is to be used (safety enhancement, driver correction, fuel consumption reduction, etc.) different parameters are used. The parameters that have been used to classify driving styles are shown in Table 2.

Driving event is understood as manoeuvres occurring during the driving task, such as: acceleration, deceleration, turning or lane change, which can be used to identify driving style. The driving events that have been studied are braking [18,37], car-following [16], distance keeping [35], roundabouts [24], lane change and cornering [9,16,18].

There are numerous approaches to classifying driving styles. The revision to the literature reveals a prevalence of simple classification bases using either two or three categories. The distinction between aggressive and non-aggressive style was used in [9,19,21,26]. The division into three classes includes soft, normal and aggressive by Syed et al. [33], comfortable, normal and sporty as described by Dörr et al. [17], aggressive, mild and moderate styles, as defined by Xu and Deng et al. [36,37], or aggressive, conservative and moderate by Chen et al. [38]. Moderate drivers are described as an in-between group showing both aggressive and calm properties, but not conclusively belonging to either. In contrast to the previous, Constantinescu et al. [23] suggested five categories of “aggressiveness”: from non-aggressive to very aggressive, while Murphey et al. [15] proposed four different categories to classify driving styles: calm driving, normal driving, aggressive driving and “no speed”.

## 3. Materials and Methods

This section explains the methodology that has been carried out for the proper development of the experimental phase, as well as the instrumentation used in it. The procedure followed in the performance of the dynamic tests will be detailed in the next sections. The boundary condition definitions are also included.

### 3.1. Instrumented Vehicle

**Figure 1:**
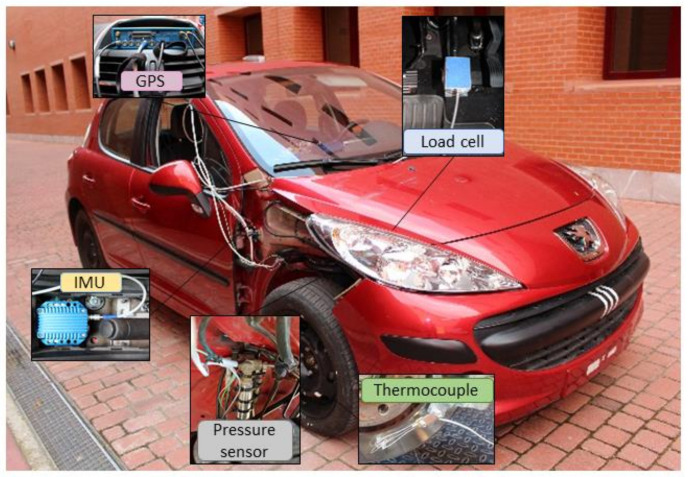
Instrumented vehicle.

The vehicle that has been used for the experimental tests is a commercial Peugeot 207 1.6 HDI 16v (see Figure 1). The vehicle’s tare weight is 1197 kg, the wheelbase 2540 mm, the height of the centre of gravity (CoG) is 410 mm, the lateral position (CoG) is 874 mm and the longitudinal position (CoG) is 1484,74 mm, measured from the front of the vehicle. The service braking system is a four-wheel hydraulic system with two independent circuits (type *X*). It uses disc brakes on both axles and has Anti-lock Braking System (ABS) and Electronic Stability Program (ESP) assistance systems.

The sensors installed on the vehicle to obtain the required dynamic parameters are described in the next subsections.

#### 3.1.1. Pressure Sensor

**Figure 2:**
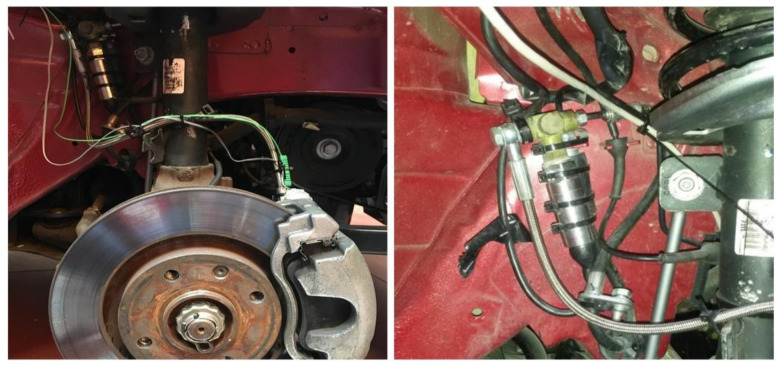
Pressure sensor in the brake circuit.

The pressure sensor was installed in the hydraulic circuit of the braking system with the aim to determinate the braking capacity of the system. In order to measure the pressure as close as possible to the brake calliper system, the pressure sensor was fixed between the brake calliper inlet and the last section of the hydraulic brake circuit, with the idea of avoiding pressure fluctuations during braking. In addition, this set up ensures that there is not interfere with other parts of the vehicle (steering, suspension, etc.) (see Figure 2). It is a PDCR 911 model by Druck Limited with an operating range from 0 to 135 bar. The pressure applied to the sensor produces a deflection of the diaphragm, which flexes the full-bridge gauges, producing a measurable voltage difference proportional to the measured pressure.

**Figure 3:**
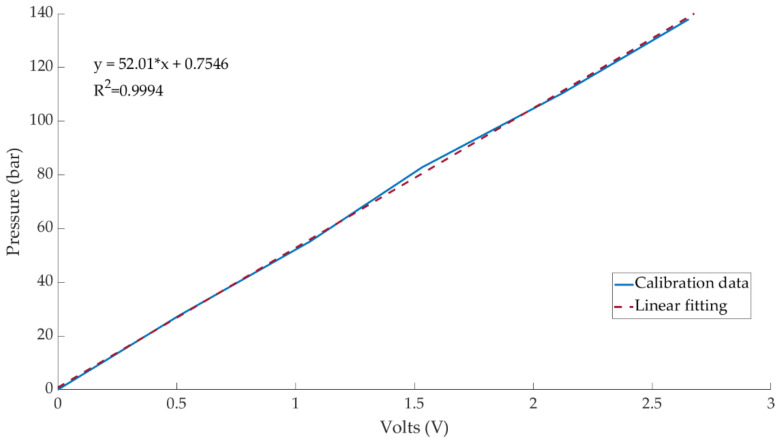
Pressure sensor calibration curve.

Once the sensor was installed in the vehicle, it was calibrated, and the calibration curve is shown in Figure 3.

#### 3.1.2. Load Cell

**Figure 4:**
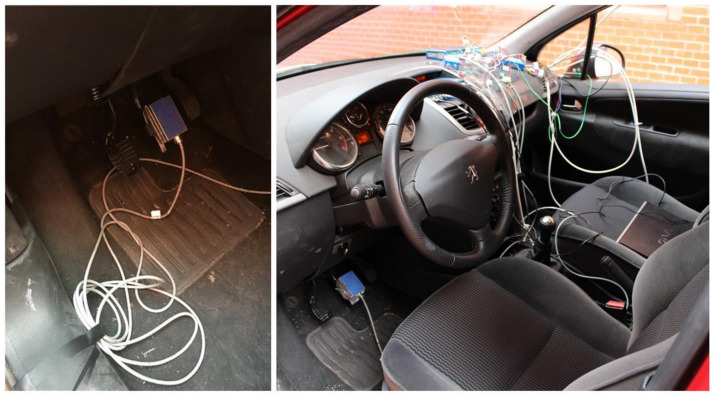
Load cell on the brake pedal.

A load cell was fixed on the braking pedal and used to measure the force applied by the driver by means of several strain gauges. The HKM PK 2.0 sensor is specially designed for this purpose (see Figure 4).

The operating range of this device is from 0 to 1500 N.

**Figure 5:**
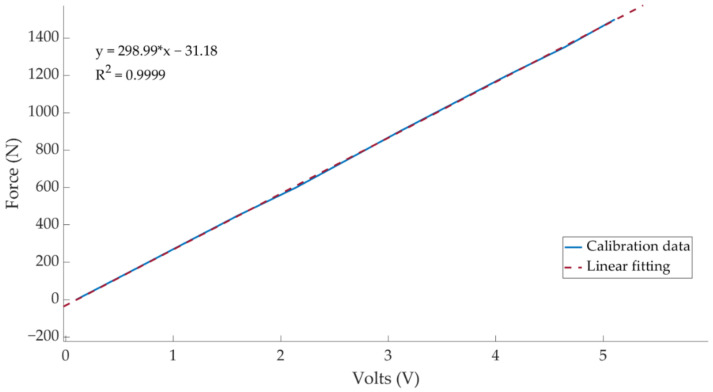
Load cell calibration curve.

Also, this sensor was calibrated before the experiments and result is shown in Figure 5:

#### 3.1.3. Inertial Measurement Unit (IMU)

The IMU was positioned at the vehicle’s CoG. The RACELOGIC IMU03 model is used to measure the longitudinal deceleration in the *X*-axis. The acceleration range is ±1.7 g.

**Figure 6:**
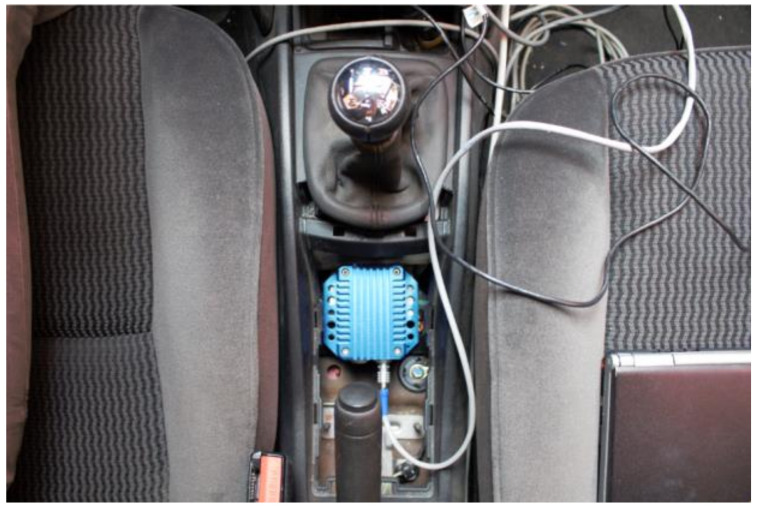
IMU installed inside the vehicle.

The IMU is permanently installed at the vehicle’s CoG, level with the ground and oriented with the main direction of movement (see Figure 6).

#### 3.1.4. Thermocouple

**Figure 7:**
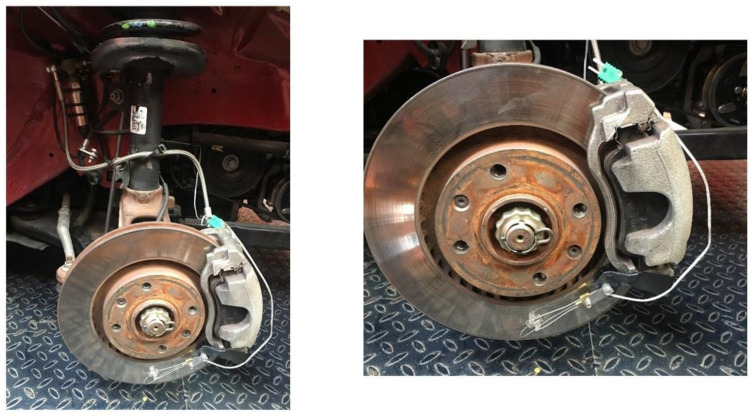
K type thermocouple installed on the brake disc.

Since dynamic tests are to be carried out on the track, the brakes reach high temperatures during operation. As is known, the temperature greatly affects the braking efficiency of the vehicle. The brake disc was instrumented to measure the temperature during the tests by installing a thermocouple K type (see Figure 7). The thermocouple is from TC DIRECT with mineral insulation of 0.5 mm diameter. The temperature range is from 0° to 850° and the time constant is 0.03 s.

#### 3.1.5. Data Acquisition System

**Figure 8:**
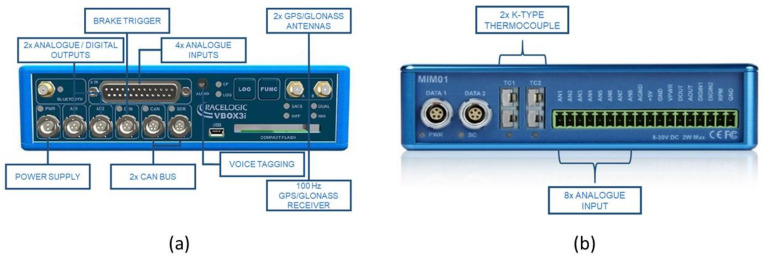
(**a**) Analogue and digital inputs and outputs available on the VBOX 3i Dual Antenna; (**b**) Inputs of the external VBOX Mini Input module.

To measure the dynamic parameters of the vehicle during the track tests, a VBOX 3i Dual Antenna data acquisition equipment is used (see Figure 8a). A CAN bus connection is used for DATA 1 of the external VBOX Mini Input module (see Figure 8b).

**Figure 9:**
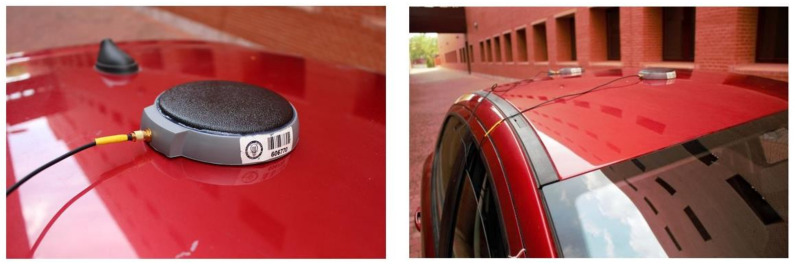
Twin antennas located longitudinally on the roof of the vehicle.

Two antennas were connected to the main VBOX 3i module. Both are longitudinally oriented on the vehicle’s roof (see Figure 9). The main antenna provides the time, speed and position values through the Doppler effect on the GPS carrier signal.

**Figure 10:**
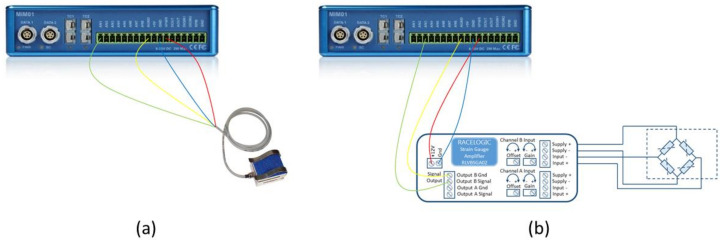
(**a**) Connection of the load cell on the brake pedal to analogue input 1 of the Mini Input; (**b**) Connection of the pressure sensor to analogue input 2 of the Mini Input.

The thermocouple is connected to TC1 input of the Mini Input module and the inertial measurement unit is connected to DATA 2. The sensors onboard the vehicle are connected to the analogue inputs of the external module. Figure 10a shows the connection of the load cell installed on the brake pedal. To fit the signal generated by the pressure sensor and be able to record by the data acquisition system, it is necessary to install an auxiliary strain gauge module. Figure 10b shows the connection of the pressure sensor to the external module utilizing full Wheatstone bridge configuration.

### 3.2. Test Methodology

This section defines the procedure for carrying out the tests and the conditions under which they were carried out.

The experimental tests are designed to reproduce all types of braking that can happen while driving a vehicle. The braking of the car is largely dependent on the brake pedal exertion. Although many different situations may arise, the most frequent frames are: when the driver maintains the brake pedal in the same position (maintained braking), when the driver slowly increases the pressure on the pedal (progressive braking) and when the driver brakes sharply (emergency braking). More information related to the braking situations is explained in the Section 3.2.1. These situations can be applied and hybridize according to the speed and desires of the driver.

All tests were carried out at the tracks of the National Institute of Aerospace Technology (INTA), which dimensions are 300 × 250 m and paves with asphalt. The driver shall perform a series of braking with the vehicle until it stops, following a straight path. The kinds of braking that the driver must performed were: maintained, progressive and emergency braking. Moreover, it is important to underline that the speed car moved between 20 and 80 km/h, increasing the speed of 10 km/h between experiments.

The tests are carried out by 14 different drivers so that the results obtained reflect the influence of each driver’s driving style. All the drivers who carried out the experimental tests are men between the ages of 22 and 30. They had driving experience from 4 to 12 years and the same level of education.

**Figure 11:**
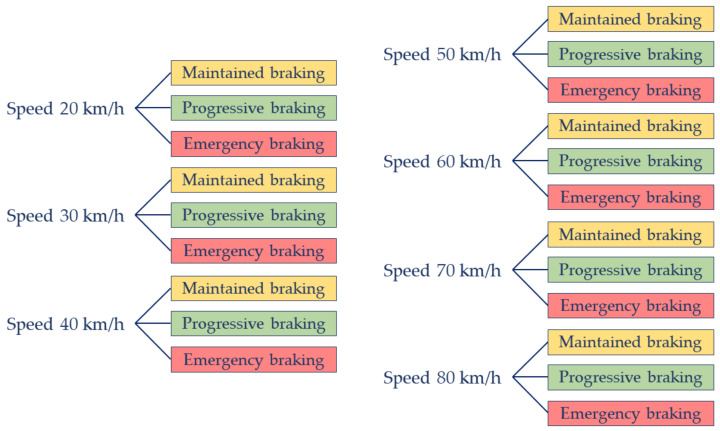
Tests carried out by different drivers.

Figure 11 shows an outline of the tests carried out by each driver. Each driver performs the three types of braking for each test speeds. Altogether 21 braking tests.

**Figure 12:**
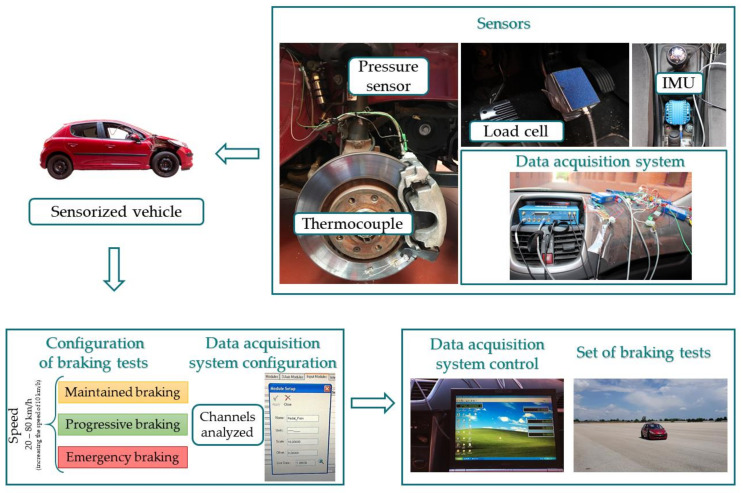
Methodology of the experimental phase.

Figure 12 shows a flow chart for the data acquisition of the experimental phase, as well as the different sensors and equipment used.

The scale and offset of each signal were adjusted. For data acquisition, a sampling frequency of 100 Hz was set. This frequency is able to register the input signal of the different installed sensors without losing information and with enough data resolution. All the data that were collected thought the test was stored to be analysed later.

#### 3.2.1. Types of Braking Performed in the Experimental Tests 

In this article, three types of braking manoeuvre are studied in order to classify different driving styles.

- Maintained braking

Maintained braking is constant and continuous over the time. It is achieved by exerting the pedal and maintaining that pressure until the vehicle stops. This type of braking is used in circulation when the braking manoeuvre can be foreseen well in advance.

- Progressive braking

Progressive braking is by a linear increase of the applied force in time. The slope determines the level of smoothness of the manoeuvre. The driver exerts a progressive pressure on the pedal. The braking force achieved increases linearly until the vehicle stops. This kind of braking is usually done when driving a vehicle.

- Emergency braking

Emergency braking is identified as being fast and hard. It is achieved by pressing the brake pedal impulsively and firmly until the end of its travel, thus causing the maximum possible braking intensity. This type of braking is done in emergencies where the driver decides to stop the vehicle in a short time.

**Figure 13:**
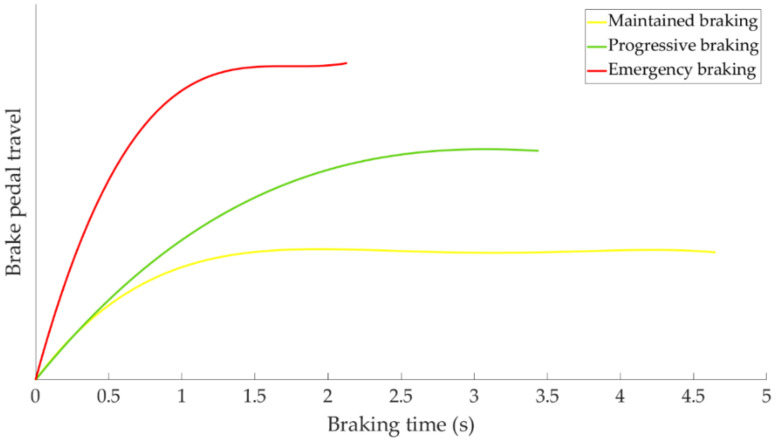
Brake pedal travel as a function of braking time for the three types of braking studied.

Figure 13 shows how the driver presses the brake pedal for each of the three types of braking.

As can be seen in the Figure 13 the maintained braking is the most “moderate” braking of the three and has been associated with a “cautious” driving style. The “usual” braking manoeuvre corresponds to progressive braking and it is considered a “normal” driving style. Finally, emergency braking is described as “hard” and it is attributed to an “aggressive” driving style.

#### 3.2.2. Variables Analysed in the Track Tests

The variables registered during the experimental braking test are described in the following lines:

1. Braking time

The braking time was enveloped between the time when the driver presses the brake pedal until the vehicle stops (speed is equal to 0 km/h).

2. Braking distance

The braking distance was defined as the distance the vehicle travelled from the time the brake pedal is pressed to the time the vehicle stops.

3. Deceleration

The longitudinal deceleration of the vehicle was collected by the inertial measurement unit (IMU).

4. Brake pedal force exerted by the driver

The force exerted by the driver on the brake pedal during the braking is gathered employing the load cell installed on the pedal (see Figure 4). This sensor has been used as a “trigger” to start the signal from the other sensors.

5. Pressure in the brake circuit

The pressure in the hydraulic system is registered by the built-in pressure sensor between the brake calliper and the last section of the hydraulic brake circuit.

#### 3.2.3. Test Conditions

To ensure the repeatability of the braking tests, the following boundary conditions were considered:

- During the tests, the pressure of the tyres must be checked according to the manufacturer’s instructions.
- The temperature on the brake disc, before starting any braking test, shall be within 18 and 31 °C. If the temperature was higher, the disc shall be cooled down before starting a new braking test. High disc temperatures could affect negatively to the braking efficiency.
- In addition to the driver, a second person in the co-driver’s seat shall be responsible for controlling the data acquisition system in all tests and monitoring the test conditions.
- All tests shall be carried out with the clutch disengaged, so that the engine’s holding power do not affect the braking capacity.

## 4. Results

In this point the results obtained in the experimental tests for each condition of speed and type of braking preset for the execution of the same are analysed. The results are presented in three blocks. Section 4.1 contains the results of the different sensors during the experimental tests. In Section 4.2, a statistical study is carried out with all the recorded data and Section 4.3 proposes a classifications of driving styles.

### 4.1. Output Signal of the Different Sensors Installed for the Driving Braking Tests

**Figure 14:**
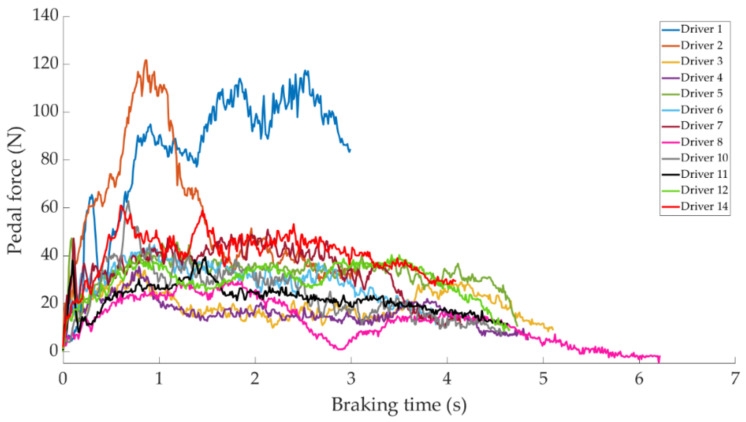
Brake pedal force curves obtained by the load cell during maintained braking at a speed of 80 km/h for different drivers.

**Figure 15:**
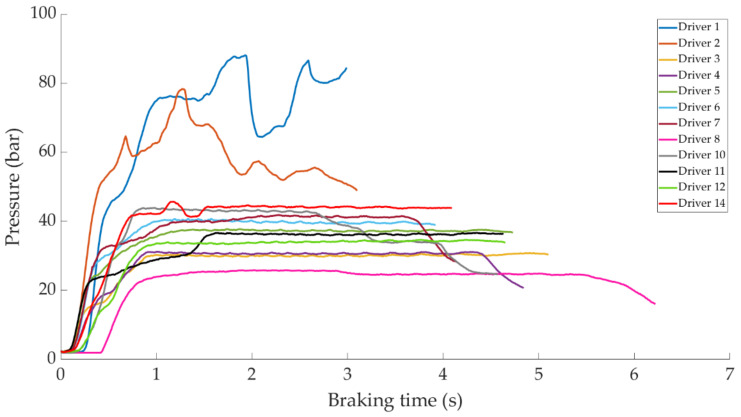
Curves of the pressure obtained in the pressure sensor during maintained braking at a speed of 80 km/h for the different drivers.

**Figure 16:**
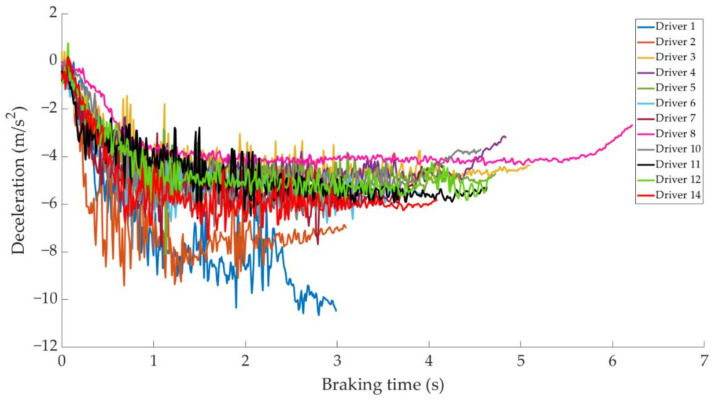
Vehicle deceleration curves obtained by the IMU during maintained braking at a speed of 80 km/h for different drivers.

**Figure 17:**
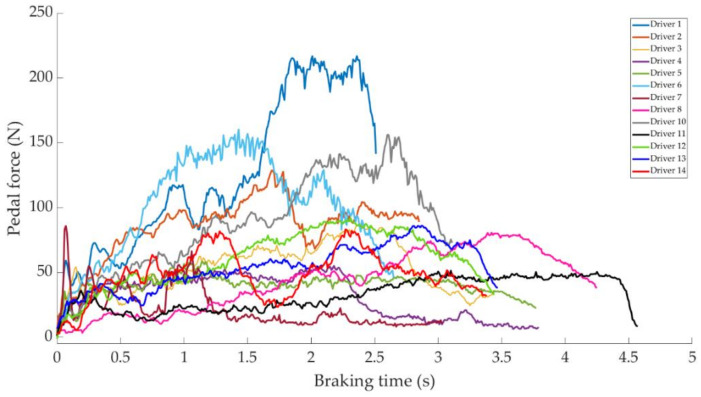
Brake pedal force curves obtained by the load cell during progressive braking at a speed of 80 km/h for different drivers.

**Figure 18:**
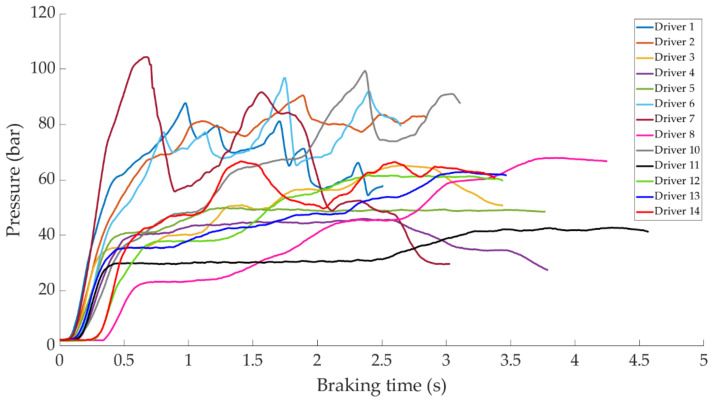
Curves of the pressure obtained at the pressure sensor during progressive braking at 80 km/h for different drivers.

**Figure 19:**
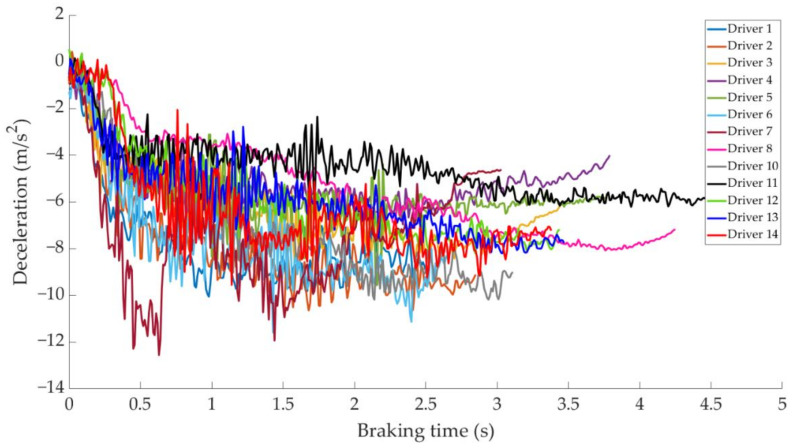
Vehicle deceleration curves obtained by the IMU during progressive braking at a speed of 80 km/h for different drivers.

**Figure 20:**
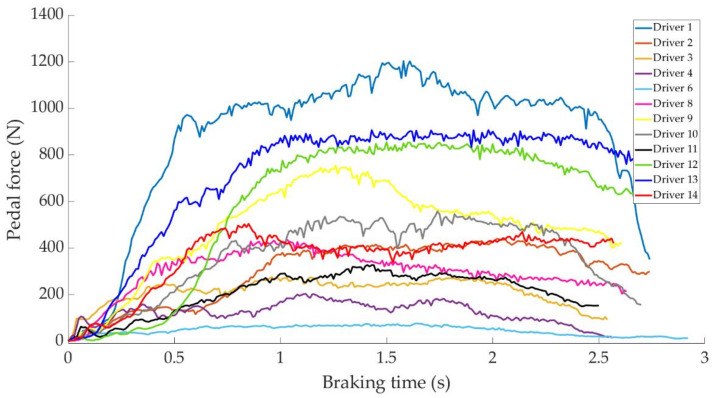
Brake pedal force curves obtained by the load cell during emergency braking at 80 km/h for different drivers.

**Figure 21:**
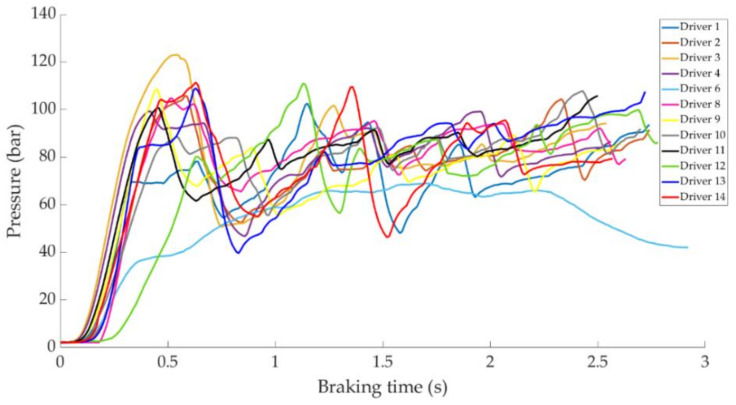
Curves of pressure obtained at the pressure sensor during emergency braking at 80 km/h for different drivers.

**Figure 22:**
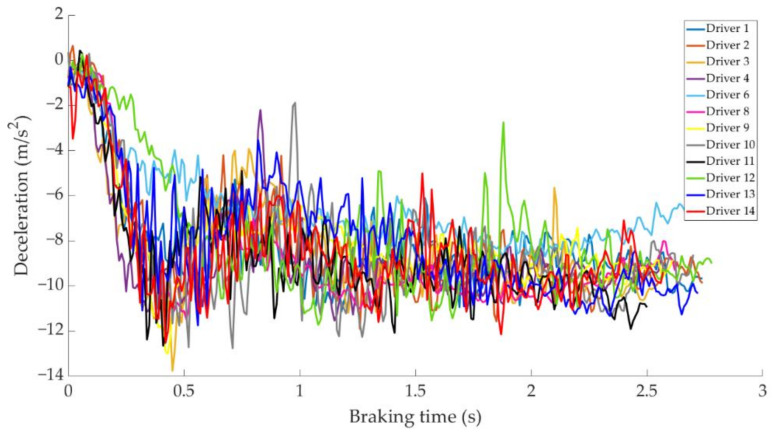
Vehicle deceleration curves obtained by the IMU during emergency braking at a speed of 80 km/h for different drivers.

In this section it is analysed how the driver performs the braking and how it is reflected in the different parameters studied. Some examples of the load cell, pressure sensor and IMU regarding to 80 km/h braking tests are shown under these lines. It is important to underline that all the information presented in this point is based on raw data from the sensors. Moreover, it is relevant to explain that those figures (Figure 14, Figure 15, Figure 16, Figure 17, Figure 18, Figure 19, Figure 20, Figure 21 and Figure 22) represent the individual behaviour of each driver and each driver is greatly affected by different factors. In any case, all of the figures provide relevant information on the variability that exists between the drivers, that can be seen in the data recorded through the time of braking. In addition, as it is going to be realized in the coming sections, the curves represent how the type of braking influences the magnitude and trend of the acquired data.

Figure 14 shows a comparison of the curves relating to the force exerted by drivers on the brake pedal for maintained braking. It can be seen that drivers 1 and 2 exert greater forces on the brake pedal than other drivers, with maximum values of around 120 N. Driver 14 is the next driver to register the higher forces on the pedal, with the maximum value being 60 N.

Figure 15 shows a comparison of the curves corresponding to the pressure obtained by the pressure sensor for the different drivers while maintained braking. Drivers 1 and 2 behave similar to each other, but different from the others. These drivers have not executed the braking maintained correctly and obtain a maximum pressure value of 90 and 80 bar, respectively. The rest of the drivers acquire pressures between 24 and 45 bar (in the constant braking section), of which the minimum corresponds to driver 8 and the maximum to driver 14.

Figure 16 shows a comparison of the curves corresponding to the deceleration suffered by the vehicle during maintained braking for different drivers. The minimum deceleration is −3.9 m/s^2^ which is achieved by driver 8 and the maximum deceleration is reached by driver 1 of −10.8 m/s^2^, followed by that recorded by driver 2 of −9.8 m/s^2^.

Figure 17 shows a comparison of the curves corresponding to the force exerted by different drivers on the brake pedal to develop progressive braking. The significant differences in the values recorded by each driver can be seen. Drivers 1, 6 and 10 are the ones who apply the most force to the brake pedal.

Figure 18 shows a comparison of the curves corresponding to the pressure obtained by the pressure sensor for the different drivers during progressive braking. Driver 7 is the one that reaches the maximum pressure of 108 bar.

Figure 19 shows a comparison of the curves corresponding to vehicle deceleration for different drivers in a progressive type of braking. Driver 7 is the one with the steepest deceleration curve and obtains a maximum value of −12 m/s^2^.

Figure 20 shows a comparison of the curves corresponding to the force exerted by the different drivers on the brake pedal to develop an emergency type of braking. A large difference can be seen between driver 6, who exerts the least force on the brake pedal, and driver 1, who exerts the most. The difference is 1110 N.

Figure 21 shows a comparison of the curves corresponding to the pressure obtained by the pressure sensor for the different drivers during emergency braking. The curve relating to driver 6 does not show the same pattern with respect to the rest of the curves, as can be seen in Figure 20, this driver exerts too little force on the brake pedal for emergency braking. Driver 3 records the maximum pressure value of 125 bar.

Figure 22 shows a comparison of the curves corresponding to vehicle deceleration during emergency braking for different drivers. The maximum deceleration value recorded is −13.76 m/s^2^ by driver 3.

### 4.2. Statistical Study of the Driver Set

In this section, a statistical study of all the variables measured during the 267 experimental tests is presented. Section 4.2.1 analyses the data obtained by means of the load cell, pressure sensor and IMU, and Section 4.2.2 analyses the data collected using GPS positioning.

#### 4.2.1. Statistical Study of the Variables: Brake Pedal Force Exerted by the Driver, Pressure in the Brake Circuit and Deceleration

The following tables show information of the 14 drivers in every test speed (maximum, minimum, average and standard deviation recorded by different sensors). The data related to maintained braking is shown in Table 3, the data related to progressive braking in Table 4 and finally, the data related to emergency braking in Table 5.

Table 3, Table 4 and Table 5 show, for the three types of braking, that the average of all the signals increases as the test speed increases. For the same test speed, the highest values recorded by the sensors are given for emergency braking, followed by progressive braking and the lowest values correspond to maintained braking.

#### 4.2.2. Statistical Study of the Following Variables: Braking Time and Braking Distance

**Figure 23:**
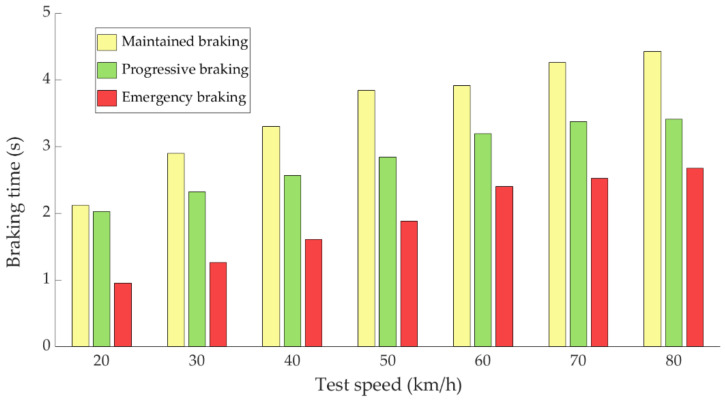
Evolution of braking time as a function of test speed for the three types of braking.

**Figure 24:**
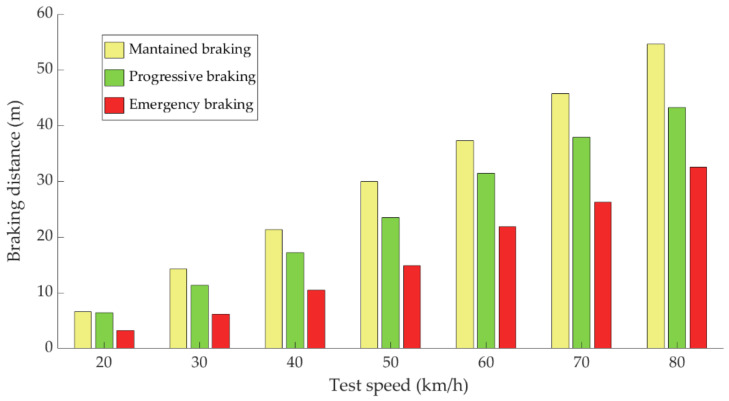
Evolution of braking distance as a function of test speed for the three types of braking.

During the experimental tests, the braking time and braking distance were also measured using GPS positioning. The results shown in Figure 23 and Figure 24 correspond to the average data recorded by the 14 drivers who carried out the experimental tests. The braking time as a function of the test speed and the type of braking can be seen in Figure 23.

Figure 23 shows that emergency braking is positioned as the fastest, followed by progressive braking and the slowest braking is the maintained braking. This data is directly related to the braking distance required for each of the braking types. As expected, the braking time depends on the type of braking.

Figure 24 shows the evolution of the braking distance as a function of the test speed and the type of braking.

Figure 24 shows that the braking distance increases as the test speed increases. Maintained braking leads to a greater distance travelled until the vehicle comes to a complete stop, followed by progressive braking and finally emergency braking.

### 4.3. Classification of Driving Style

In this section, classifications are proposed for the three driving styles: cautious, normal and aggressive. The classifications are established as a function of vehicle speed and independently for the pressure in the brake circuit, the force exerted by the driver on the brake pedal, vehicle deceleration and for braking power.

Section 4.3.1 explains the methodology followed for the analysis of the data collected during the experimental tests and which allows the classifications shown in Section 4.3.2 to be made.

#### 4.3.1. Data Analysis Methodology

**Figure 25:**
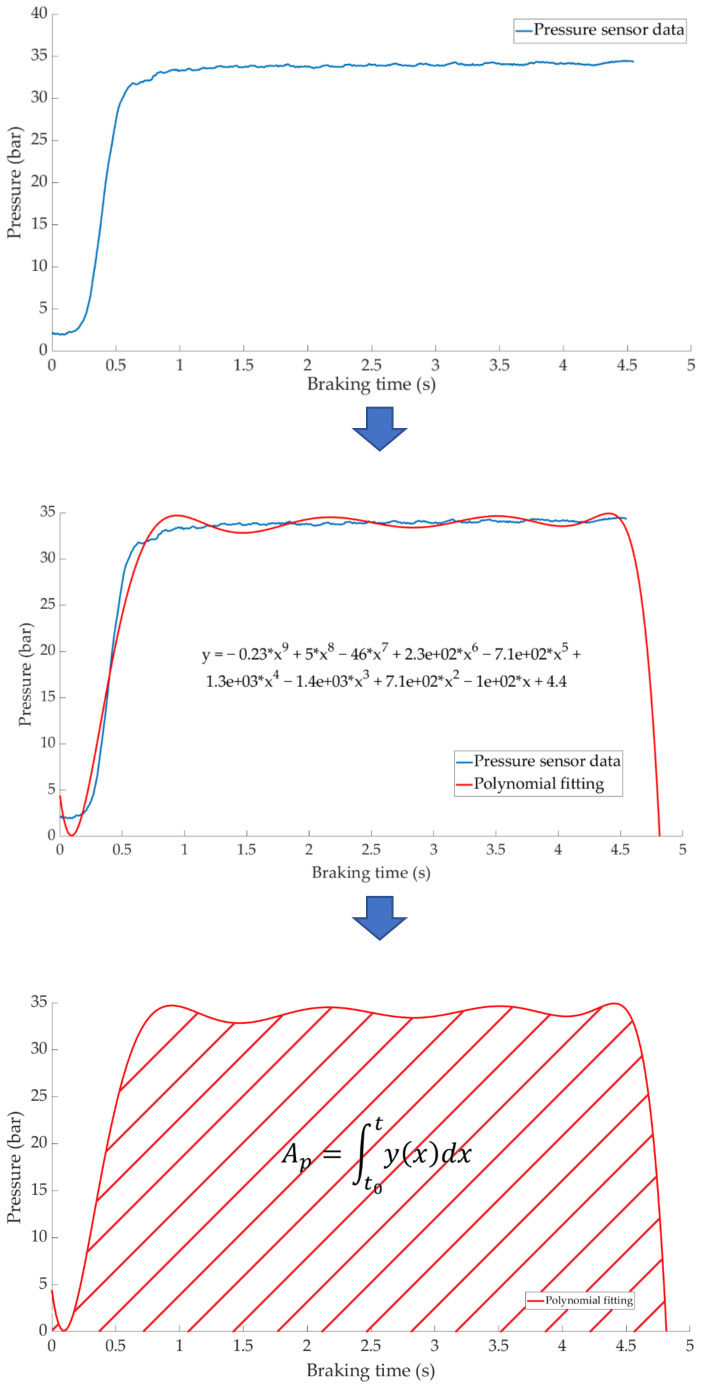
Methodology for the analysis of the data acquired by the various sensors.

The data analysis methodology to perform the classification of the different types of braking consists of calculating the area enclosed under the curves of pressure in the brake circuit, force exerted by the driver, vehicle deceleration and kinetic energy. The first step is to fit the curves representing the temporal evolution of the measurements obtained by the sensors during each braking to a polynomial. Once the fitting function is known, it is possible to calculate the area enclosed under the curve. The area is defined as the integral of the fitted function from the start of braking to the end of braking and provides significant information regarding the magnitude of the braking (see Figure 25).

#### 4.3.2. Classification of the Types of Braking for Different Variables

The values recorded by the various sensors during the braking time are used to classify the different types of braking. For each type of braking and speed, the average of the areas corresponding to the 14 drivers has been calculated.

Knowing the test speed and the curve area of the parameters described above, it is possible to identify the type of braking for a new manoeuvre.

**Figure 26:**
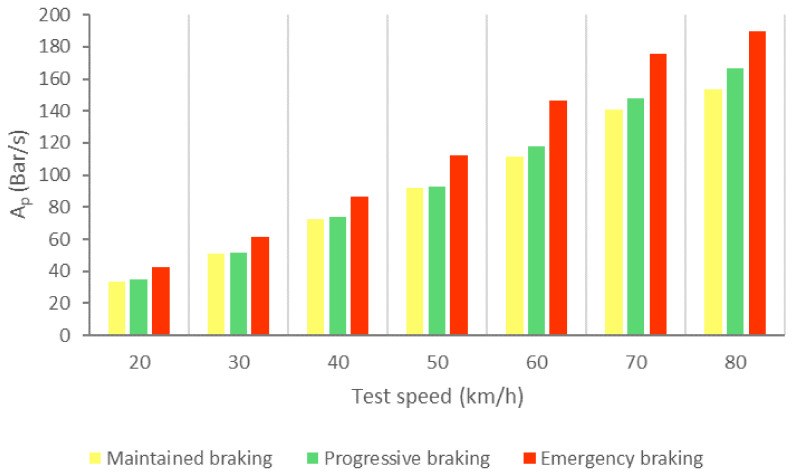
A_p_ for the three types of braking and different test speeds.

Figure 26 shows the calculated average area of the pressure in the brake circuit (A_p_) for the three types of braking and different test speeds.

Table 6 shows the intervals of the areas that allow us to identify which type of braking is being performed as a function of the circulation speed and shows the classification according to the data collected by the pressure sensor.

**Figure 27:**
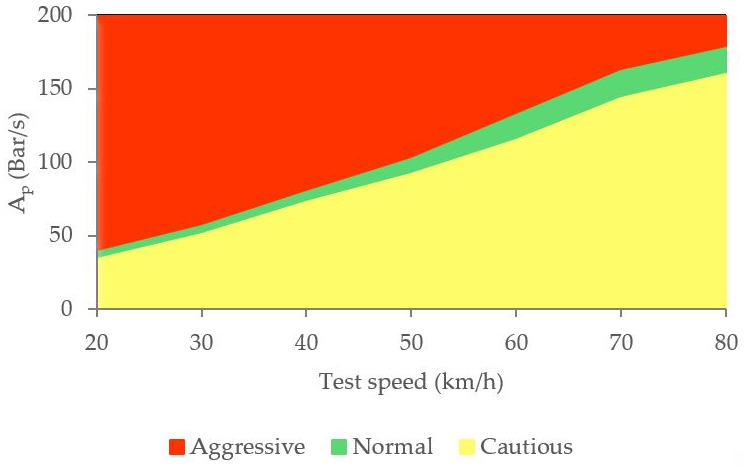
Classification of driving styles by pressure in the brake circuit.

A more visual representation of Table 6 is shown in Figure 27. Figure 27 allows three driving styles to be identified: cautious, normal and aggressive using vehicle speed and pressure sensor information.

**Figure 28:**
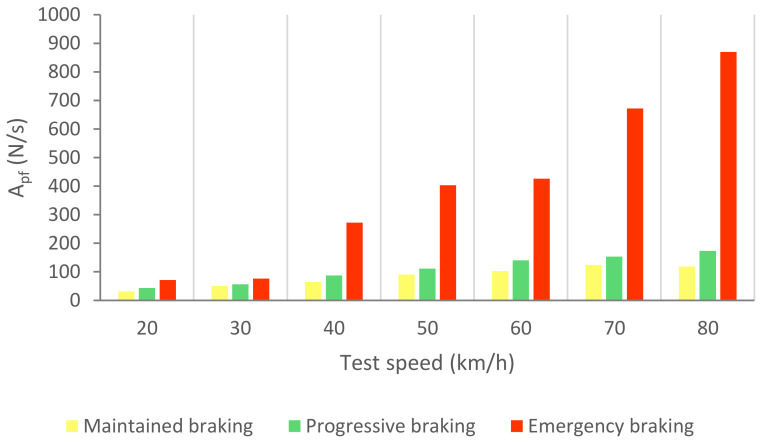
A_pf_ for the three types of braking and different test speeds.

Figure 28 shows the calculated average area related to the brake pedal force curves (A_pf_) for the three kinds of braking and different test speeds.

Table 7 shows the area ranges that allow us to identify the different types of braking based on the data collected by the load cell.

**Figure 29:**
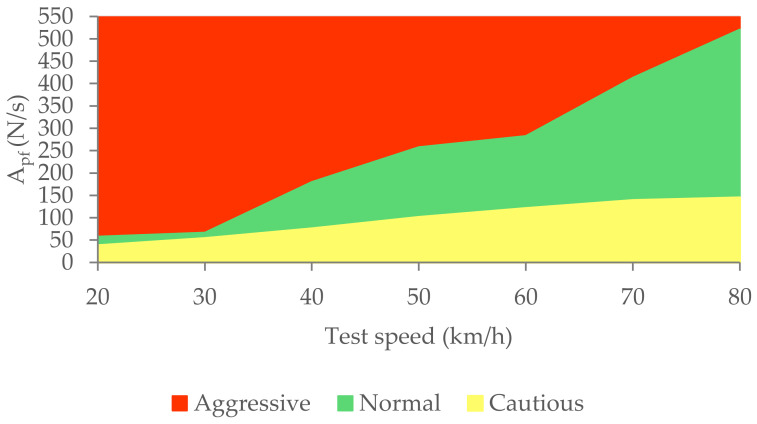
Classification of driving styles by the force the driver exerts on the brake pedal.

Based on the circulation speed and the information provided by the load cell, the classification of driving styles can be seen in Figure 29.

**Figure 30:**
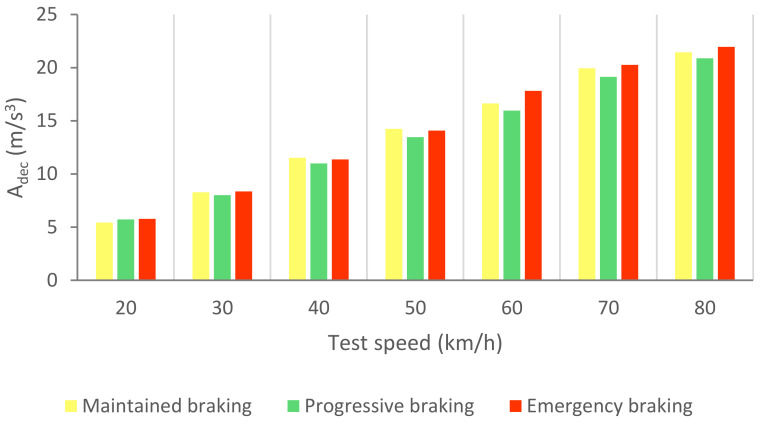
A_dec_ for the three types of braking and different test speeds.

Figure 30 shows the calculated average area relating to the deceleration curves (A_dec_) for the three types of braking and different test speeds.

Table 8 shows the gaps in the areas that enable us to define the different types of braking by means of the longitudinal deceleration of the vehicle.

**Figure 31:**
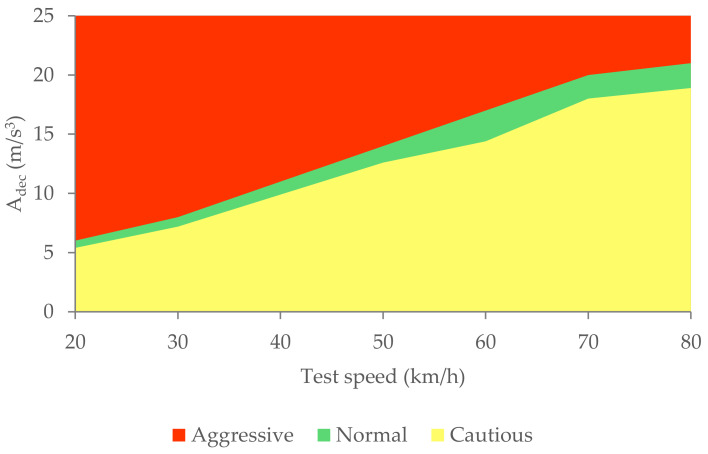
Classification of driving styles by longitudinal vehicle deceleration.

The classification of driving styles based on vehicle speed and deceleration is shown in Figure 31.

**Figure 32:**
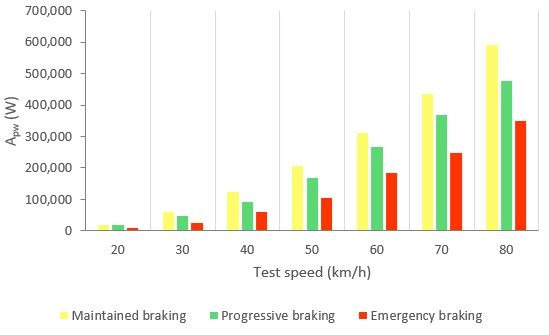
A_pw_ for the three types and different test speeds.

Figure 32 shows the calculated average area relating to the kinetic energy curves (A_pw_) for the three types of braking and different test speeds. The kinetic energy (*E_k_*) of the braking system is determined with Equation (1). The instantaneous vehicle speed (*V*) is known from the GPS and *m* is the tare weight of the vehicle and the mass of the two occupants.

(1) $$E_{k}=\frac{1}{2}mV^{2},$$

The integral of the kinetic energy curve between the time limits makes possible to assess the power developed by the brake. In Table 9 it is shown the average braking power in each kind of braking manoeuvre and test speeds for all 14 drivers. The power increases as the test speed increases and according to the type of braking in the following order: maintained, progressive and emergency.

**Figure 33:**
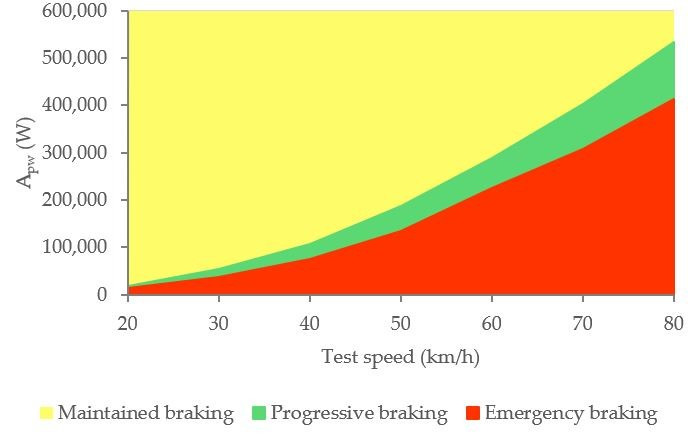
Classification of driving styles by vehicle kinetic energy.

Figure 33 shows the classification of driving styles with knowledge of the vehicle’s instantaneous speed.

## 5. Discussion and Conclusions

This article presents a successful methodology to classify different driving styles according to the braking manoeuvre and through the readings obtained by different sensors placed in strategically studied points of a vehicle. A pressure sensor has been installed in the independent hydraulic circuit of the front right wheel and a load cell mounted on the brake pedal. A GPS receiver and an IMU have also been mounted on the car. The efficiency of these sensors for taking highly accurate measurements during dynamic tests has been demonstrated.

Three types of braking have been proposed, which are intended to cover all driving circumstances in which the braking system may be involved: maintained, progressive and emergency. These types of braking have made it possible to define three driving styles. Maintained type braking is achieved by exerting a single pressure on the brake pedal and maintaining it until the vehicle stops. For progressive braking, the driver gradually increases the pressure on the brake pedal until the vehicle stops. Finally, emergency braking is achieved by firmly pressing the brake pedal until the end of its travel. In that way, maintained braking is the most “moderate” of the three and has been associated with a “cautious” driving style. The “usual” braking manoeuvre corresponds to progressive braking and is considered a “normal” driving style. Finally, emergency braking is described as “hard” and is attributed to an “aggressive” driving style.

Experimental tests have been carried out for driving on a straight line on flat ground for each type of braking: maintained, progressive and emergency; for a range of speeds between 20 and 80 km/h, increasing the speed in 10 km/h intervals. The driver moves forward with the vehicle until the test speed is reached and at the moment when the speed is constant, the braking process begins.

The data acquired in the experimental tests made it possible to satisfactorily relate the driving speed, the type of braking and the manner in which the driver performs the braking manoeuvre to the reading of the various sensors on board the vehicle. The direct readings of the different sensors for the different test conditions have provided relevant information and have confirmed that the parameters chosen to characterise the driver’s behaviour are appropriate. The figures shown in Section 4.1 demonstrate that each driver has his own driving style when performing any of the three types of braking manoeuvre. The type of braking for which drivers differ the most is emergency braking. Figure 20 shows how drivers apply forces of different magnitudes to the brake pedal. Figure 18, Figure 19 and Figure 20, for progressive braking, show that drivers 1, 2, 6, 7, 10 and 14 apply more force to the brake pedal at the start of braking to reduce speed immediately and then apply a lighter force until the vehicle stops, than drivers 3, 4, 5, 8, 11, 12 and 13 who apply a lighter brake pedal at the start of braking and then gradually apply more force until the vehicle is stopped. The maintained type of braking is the one in which the least differences are seen between the different drivers, see Figure 14, Figure 15 and Figure 16, excluding drivers 1 and 2 who do not maintain pressure on the brake pedal to perform a braking of this type correctly. Table 3, Table 4 and Table 5 show the increasing average of the values recorded by each sensor due to the increase in speed, for the three types of braking. For the same test speed, the minimum values collected correspond to maintained type braking, followed by progressive type braking and, finally, the maximum values are those recorded for emergency braking.

The three types of braking studied have been characterised thanks to the analysis of the data collected by the different sensors during the experimental tests. The methodology used to analyse the data consists of calculating the area enclosed under the curves corresponding to the pressure in the brake circuit, the force exerted by the driver on the brake pedal, the longitudinal deceleration of the vehicle and the kinetic energy for the duration of the braking. A classification of the three types of braking is established for each of these variables independently according to vehicle speed. Table 6, Table 7, Table 8 and Table 9 show the intervals of the areas that allow the identification of the type of braking performed for each parameter as a function of speed. Table 6 shows the classification based on the pressure in the hydraulic brake circuit. Table 7 shows the classification according to the force exerted by the driver on the brake pedal. The classification related to vehicle deceleration and braking power is shown in Table 8 and Table 9, respectively. The aim of this article, which was to classify different driving styles based on the proposed types of braking, has been achieved. These classifications are shown in Figure 27, Figure 29, Figure 31 and Figure 33. In these figures, depending on the vehicle speed for the pressure in the brake circuit, the force exerted by the driver on the brake pedal, the longitudinal deceleration of the vehicle and the kinetic energy, the following driving styles can be determined: cautious, normal and aggressive. These figures make it possible to identify a driver’s driving style for any braking manoeuvre by knowing the vehicle speed and the area under the curve of one of the above variables.

In future developments, the authors aim for a vehicle to be able to reproduce the habits and ways of acting of drivers during a braking manoeuvre, but correcting possible human errors linked to distractions, lack of visibility or reaction times. To achieve this purpose and with the analysis of the data obtained in this study, an estimation system based on Artificial Neural Networks will be created, which will try to forecast the behaviour of the different systems according to the boundary conditions that regulate the braking manoeuvre. The system will be able to simulate the real results collected by the sensors in order to characterize any type of braking and thus be used in real conditions of circulation. Specifically, the system will simulate the pressure in the hydraulic brake circuit, the longitudinal deceleration of the vehicle and the force exerted by the driver on the brake pedal. Using controllers, this system could be implemented in autonomous vehicles in such a way that the user can choose the driving style that best suits his perception of comfort.

## Figures and Tables

**Table 1:** Sensors required for the study of driving styles.

Sensor	Reference
IMU	[15,16,17,18]
Low-cost accelerometers	[21]
Smartphone	[9,19,20,22]
GPS	[23,24]
GPS and Inertial	[25]
Radar or LiDAR	[17,26,27]

**Table 2:** Measured parameters for the study of driving styles.

Parameters	Reference
Power/fuel demand/consumption	[22,28,29,30]
Acceleration	[18,20,25,26,31,32,33,34]
Speed	[25,31,32,33,34,35,36]
Deceleration	[31,32]
Gas pedal position	[16,32,33,35,36]
Brake pedal position	[16,35]
Jerk	[15,26]
Yaw/pitch/roll	[18,20]
Brake pressure	[36]
Distance-keeping to lead vehicle	[26,35]
Engine speed/wheel rotation/gear-changing behaviour	[32]

**Table 3:** Data from the different sensors for each test speed for maintained braking.

Speed (km/h)	Sensor	Maximum	Minimum	Average	Standard Deviation
20	Pedal force (N)	26.97	17.15	23.64	2.89
	Pressure (Bar)	40	11.45	22.77	9.08
	Deceleration (m/s^2^)	−6.10	−2.19	−3.92	1.27
30	Pedal force (N)	29.56	23.09	27.07	1.84
	Pressure (Bar)	41.94	14.47	24.56	8.98
	Deceleration (m/s^2^)	−7.32	−2.99	−4.42	1.34
40	Pedal force (N)	44.55	20.25	31.01	8.18
	Pressure (Bar)	42.35	17.84	29.33	8.52
	Deceleration (m/s^2^)	−7.89	−3.15	−5.23	1.47
50	Pedal force (N)	47.87	24.13	33.66	7.01
	Pressure (Bar)	61.37	15.77	32.65	12.35
	Deceleration (m/s^2^)	−8.84	−3.22	−5.62	1.69
60	Pedal force (N)	56.19	29.56	38.14	7.04
	Pressure (Bar)	73.96	19.28	35.46	13.84
	Deceleration (m/s^2^)	−10.43	−3.70	−6.40	1.92
70	Pedal force (N)	53.34	30.33	43.59	7.99
	Pressure (Bar)	83.23	21.03	40.71	15.20
	Deceleration (m/s^2^)	−10.25	−3.92	−6.66	1.64
80	Pedal force (N)	121.85	29.56	57.26	30.80
	Pressure (Bar)	88.09	25.89	44.62	19.03
	Deceleration (m/s^2^)	−10.66	−4.52	−7.35	1.69

**Table 4:** Data from the different sensors for each test speed for progressive braking.

Speed (km/h)	Sensor	Maximum	Minimum	Average	Standard Deviation
20	Pedal force (N)	39.90	25.12	31.35	4.19
	Pressure (Bar)	54.18	18.15	28.80	9.38
	Deceleration (m/s^2^)	−7.24	−3.12	−4.44	1.24
30	Pedal force (N)	68.85	30.85	45.43	10.39
	Pressure (Bar)	49.10	22.52	34.03	7.81
	Deceleration (m/s^2^)	−7.48	−4.35	−5.99	1.01
40	Pedal force (N)	77.13	34.99	50.68	14.05
	Pressure (Bar)	61.96	28.86	43.25	11.53
	Deceleration (m/s^2^)	−9.14	−4.51	−6.86	1.43
50	Pedal force (N)	85.14	43.00	64.27	15.61
	Pressure (Bar)	70.91	25.44	48.86	14.41
	Deceleration (m/s^2^)	−9.54	−5.57	−7.71	1.47
60	Pedal force (N)	156.70	48.17	83.98	30.64
	Pressure (Bar)	99.82	34.12	58.28	20.87
	Deceleration (m/s^2^)	−10.86	−5.44	−8.27	1.74
70	Pedal force (N)	154.68	57.74	97.90	33.50
	Pressure (Bar)	104.82	38.75	69.50	24.81
	Deceleration (m/s^2^)	−11.20	−5.66	−8.80	1.86
80	Pedal force (N)	216.93	51.53	104.06	48.44
	Pressure (Bar)	104.37	42.75	72.46	21.04
	Deceleration (m/s^2^)	−12.56	−6.49	−9.26	1.78

**Table 5:** Data from the different sensors for each test speed for emergency braking.

Speed (km/h)	Sensor	Maximum	Minimum	Average	Standard Deviation
20	Pedal force (N)	192.94	56.19	129.51	50.02
	Pressure (Bar)	118.22	40.24	81.04	28.33
	Deceleration (m/s^2^)	−12.54	−6.70	−10.03	1.87
30	Pedal force (N)	240.51	65.49	138.20	58.50
	Pressure (Bar)	121.86	53.32	88.24	25.69
	Deceleration (m/s^2^)	−13.02	−7.30	−10.66	1.74
40	Pedal force (N)	359.95	66.01	208.51	100.27
	Pressure (Bar)	112.10	52.60	94.07	16.42
	Deceleration (m/s^2^)	−12.77	−8.58	−10.94	1.19
50	Pedal force (N)	657.25	78.94	281.42	190.83
	Pressure (Bar)	113.63	65.02	95.89	13.63
	Deceleration (m/s^2^)	−12.08	−10.14	−11.24	0.67
60	Pedal force (N)	669.66	111.77	337.53	217.34
	Pressure (Bar)	105.49	77.52	98.28	7.74
	Deceleration (m/s^2^)	−12.47	−9.67	−11.36	0.76
70	Pedal force (N)	1157.49	139.69	523.00	335.60
	Pressure (Bar)	117.54	92.72	104.74	7.72
	Deceleration (m/s^2^)	−12.62	−10.57	−11.95	0.57
80	Pedal force (N)	1203.76	76.35	545.19	328.30
	Pressure (Bar)	123.03	69.02	104.84	12.70
	Deceleration (m/s^2^)	−13.76	−8.99	−11.95	1.20

**Table 6:** Classification of braking types according to pressure in the brake circuit.

Vehicle Speed (km/h)	Maintained Braking		Progressive Braking		Emergency Braking	
	From (Bar/s)	To (Bar/s)	From (Bar/s)	To (Bar/s)	From (Bar/s)	To (Bar/s)
20	0	34	34	39	39	<
30	0	51	51	57	57	<
40	0	73	73	80	80	<
50	0	92	92	102	102	<
60	0	115	115	132	132	<
70	0	144	144	162	162	<
80	0	160	160	178	178	<

**Table 7:** Classification of braking types according to the force exerted by the driver on the brake pedal.

Vehicle Speed (km/h)	Maintained Braking		Progressive Braking		Emergency Braking	
	From (N/s)	To (N/s)	From (N/s)	To (N/s)	From (N/s)	To (N/s)
20	0	38	38	58	58	<
30	0	54	54	67	67	<
40	0	76	76	180	180	<
50	0	101	101	258	258	<
60	0	121	121	283	283	<
70	0	139	139	413	413	<
80	0	145	145	521	521	<

**Table 8:** Classification of braking types according to the longitudinal deceleration of the vehicle.

Vehicle Speed (km/h)	Maintained Braking		Progressive Braking		Emergency Braking	
	From (m/s^3^)	To (m/s^3^)	From (m/s^3^)	To (m/s^3^)	From (m/s^3^)	To (m/s^3^)
20	0	5	5	6	6	<
30	0	7	7	8	8	<
40	0	10	10	11	11	<
50	0	12	12	14	14	<
60	0	14	14	17	17	<
70	0	18	18	20	20	<
80	0	19	19	21	21	<

**Table 9:** Classification of braking types according to kinetic energy.

Vehicle Speed (km/h)	Emergency Braking		Progressive Braking		Maintained Braking	
	From (W)	To (W)	From (W)	To (W)	From (W)	To (W)
20	0	13729	13729	18029	18029	<
30	0	37572	37572	54705	54705	<
40	0	75651	75651	106716	106716	<
50	0	135472	135472	187024	187024	<
60	0	225396	225396	288994	288994	<
70	0	308420	308420	402195	402195	<
80	0	413086	413086	534199	534199	<
